# Clinical Significance of Probiotics for Children with Idiopathic Nephrotic Syndrome

**DOI:** 10.3390/nu13020365

**Published:** 2021-01-26

**Authors:** Tadashi Yamaguchi, Shoji Tsuji, Shohei Akagawa, Yuko Akagawa, Jiro Kino, Sohsaku Yamanouchi, Takahisa Kimata, Masaki Hashiyada, Atsushi Akane, Kazunari Kaneko

**Affiliations:** 1 Department of Pediatrics, Kansai Medical University, Osaka 573-1010, Japan; tadashipuriusu@gmail.com (T.Y.); tsujis@hirakata.kmu.ac.jp (S.T.); akagawas@hirakata.kmu.ac.jp (S.A.); yuko611203@gmail.com (Y.A.); kinojr.co.jp@gmail.com (J.K.); yamanous@hirakata.kmu.ac.jp (S.Y.); kimatat@hirakata.kmu.ac.jp (T.K.); 2 Department of Legal Medicine, Kansai Medical University, Osaka 573-1010, Japan; hashiyam@hirakata.kmu.ac.jp (M.H.); akane@hirakata.kmu.ac.jp (A.A.)

**Keywords:** butyrate-producing bacteria, idiopathic nephrotic syndrome, probiotics, regulatory T cells

## Abstract

We previously reported that a decrease in butyrate-producing bacteria in the gut is a potential cause of regulatory T cell (Treg) abnormalities in children with idiopathic nephrotic syndrome (INS). Therefore, we hypothesized that administration of butyrate-producing bacteria might reduce INS relapse and the need for immunosuppressants in these patients. Twenty patients in remission from INS (median age 5.3 years, 15 boys) were enrolled in the study and assigned to receive either daily oral treatment with a preparation of 3 g *Clostridium butyricum* or no probiotic treatment. The number of relapses and requirement for immunosuppressive agents were compared between the two groups. In the probiotic treatment group, analyses of the gut microbiota and Treg measurements were also performed. Probiotic-treated patients experienced fewer INS relapses per year compared with non-probiotic-treated patients (*p* = 0.016). Further, administration of rituximab in the probiotic treatment group was significantly less frequent compared with the non-probiotic-treated group (*p* = 0.025). In the probiotic treatment group, analyses before and after probiotic treatment revealed the significant increases in the relative abundance of butyrate-producing bacteria (*p* = 0.017) and blood Treg counts (*p* = 0.0065). Thus, oral administration of butyrate-producing bacteria during INS remission may reduce the frequency of relapse and the need for immunosuppressive agents.

## 1. Introduction

Enormous numbers of commensal bacteria routinely make their homes in the intestines of animals, including humans. The human gut microbiota is a collection of microorganisms, including fungi, viruses, archaea, helminths, as well as symbiotic and pathogenic bacteria [1]. It has been estimated that the human colon contains several hundred different species of bacteria and >40 trillion microbial cells in total, which is greater than the number of eukaryotic cells in the human body, which is estimated at about 30 trillion [2]. Conventionally, analysis of the gut microbiota has been carried out using the isolation and culture method. However, many living bacteria in the gut cannot be cultured. In the 1990s, however, information on the gut microbiota increased dramatically after the introduction of techniques for molecular analysis of the 16S rRNA gene specific to bacteria [2,3]. Advances in the study of the gut microbiota have shown that it plays an essential role in maintaining health. The gut microbiota differs greatly depending on various factors both in utero and in the postnatal environment, including mode of delivery (vaginal or cesarean section), infant nutrition (breast milk or formula), antibiotic administration, and dietary content [4].

It has also become clear that dysbiosis, defined as changes in the composition of the gut microbiota, is associated with adult conditions such as inflammatory bowel disease and irritable bowel syndrome. Recently, several studies have demonstrated relationships between dysbiosis and pediatric conditions such as allergic diseases [5,6,7]. Although the involvement of intestinal dysbiosis has been investigated in chronic kidney disease in adults [8], the role of dysbiosis in pediatric idiopathic nephrotic syndrome (INS) has been investigated in only a few studies [9,10,11].

INS is characterized by the abrupt onset of massive proteinuria and accounts for approximately 90% of cases of childhood nephrotic syndrome. Though INS is relatively common (the cumulative prevalence is 16 per 100,000 children [12]), its pathogenesis remains elusive. Previously, studies have reported that regulatory T cells (Tregs), T cells secreting cytokines that act as inhibitors of inflammation, are significantly fewer at the onset of INS and speculated that Tregs may be involved in INS pathogenesis [13]. It was recently reported that butyric acid, an organic acid produced mainly by intestinal bacteria of the genus *Clostridium*, plays an important role in the differentiation and induction of Tregs [14,15]. Thus, we previously hypothesized that dysbiosis is responsible for the decrease in Tregs at the onset of INS, and we analyzed the gut microbiota of INS patients at that time. We found significantly fewer butyrate-producing bacteria in stools from patients at the onset of INS. We also found a significant reduction in butyric acid in the stools [9].

Therefore, this study aimed to correct dysbiosis in pediatric INS patients and to determine whether administration of a formulation containing *Clostridium butyricum*, a butyrate-producing bacterium, as a probiotic would reduce the rate of INS relapse and the need for immunosuppressive drugs.

## 2. Materials and Methods

### 2.1. Study Population

Twenty patients (median age 5.3 years, 15 boys and 5 girls) were diagnosed with INS after presenting to the Kansai Medical University Hospital with a chief complaint of proteinuria between January 2015 and December 2019. All patients included in this study had primary and not relapsed INS. The definition of INS onset was increased excretion of urinary protein (dipstick >3+ for at least 3 sequential days or urinary protein–creatinine ratio >2000 mg/g creatinine) and hypoalbuminemia (<2.5 g/dL). Remission was successfully induced in all patients by treatment with prednisolone according to the guidelines of the International Study of Kidney Disease in Children (ISKDC) [16] (60 mg/m^2^/day or 2 mg/kg/day for 4 weeks, followed by 40 mg/m^2^/day or 1.5 mg/kg/day for 4 weeks on alternate days). Remission was defined as normal serum albumin (>3.0 g/dL) and normal urinary protein levels (early morning urinary protein negative by dipstick or early morning urine showing a protein–creatinine ratio <0.2 g/g creatinine for 3 consecutive days) [17,18]. No immunosuppressive agents other than prednisolone were used for the induction of first remission in study patients. The criteria for administration of immunosuppressive drugs other than prednisolone at our institution are as follows: (1) prednisolone should be used for the first two relapses, while mizoribine should be used for the third and subsequent relapses after induction of remission, (2) frequent relapses (four or more per year) should be treated with cyclosporin, and (3) if remission cannot be maintained with cyclosporin or cyclosporin toxicity occurs, rituximab should be administered.

The patients were divided into two groups: 10 patients (probiotic-treated, median age 6.4 years) who took a preparation of butyrate-producing bacteria (*C. butyricum* MIYAIRI, CBM) after induction of INS remission, and 10 patients (non-probiotic-treated, median age 4.7 years) who did not. The probiotic-treated group took 3 g/day of CBM preparation (MIYA BM, Miyarisan Pharmaceutical Co., Tokyo, Japan). The preparation contains approximately 10^8^ colony forming units of CBM per gram of viable spores [19]. Oral administration of CBM was started at the end of the 8-week steroid administration period of the ISKDC protocol. CBM and immunosuppressive drugs were not used in combination, except at relapse. No immunosuppressive drugs, including prednisolone, were administered when blood and stool samples were collected.

Written informed consent for participation in this study was obtained from patients’ parents. The Research Ethics Review Board of Kansai Medical University approved this study (No. 2015127).

### 2.2. Fecal Sample Collection and 16S rRNA Sequencing

To analyze the gut microbiota, paired stool samples were collected at onset of INS and during treatment with probiotics from eight probiotic-treated children. In addition, stool samples were also obtained from 21 healthy children (median age 4.0 years). Stool samples were stored at −80 °C until analysis. The frozen stool specimens were thawed and prepared for 16S rRNA sequencing using the DNA Stool Kit (Macherey-Nagel, Düren, Germany). The seven hypervariable regions of 16S rRNA, except v1 and v5, were sequenced, and the 16S Metagenome Kit (Thermo Fisher Scientific, Waltham, MA, USA) was used for amplification. After purification, the libraries were constructed using the Ion Plus Fragment Library Kit (Thermo Fisher Scientific) and the Ion Xpress Barcode Adapters Kit (Thermo Fisher Scientific). Barcoded libraries were quantitated using an Agilent Bioanalyzer 2000 (Agilent, Santa Clara, CA, USA) and pooled at a final concentration of 30 pM per target. Emulsion polymerase chain reaction and target enrichment for template preparation were performed using the Ion Chef Instrument Kit (Thermo Fisher Scientific). Sequencing was performed using an Ion PGM sequencer (Thermo Fisher Scientific) and an Ion 318 chip (Thermo Fisher Scientific). All resulting sequence data were analyzed using Ion Reporter software (Thermo Fisher Scientific).

### 2.3. Flow Cytometric Analysis of Tregs

Whole blood samples were collected at three time points: at the onset of INS, immediately after the end of prednisolone administration following induction of initial remission, and 1.5 to 2 months after starting probiotic administration following prednisolone cessation in the probiotic-treated patients or with comparable timing in the non-probiotic-treated patients. To measure numbers of Tregs, 5 µL of fluorescein isothiocyanate-conjugated anti-CD4 antibody (Beckman Coulter, Fullerton, CA, USA) and 5 µL of cyanine-5-conjugated anti-CD25 monoclonal antibody (Beckman Coulter) were added to whole blood. After cell surface staining, the cells were washed and subjected to membrane permeabilization using IntraPrep™ reagent (Beckman Coulter). Subsequently, 5 µL of phycoerythrin-conjugated anti-FoxP3 monoclonal antibody (EXBIO, Vestec, Czech Republic) was added for intracellular staining.

At the end of the incubation, erythrocytes were lysed. Cells were washed twice with phosphate-buffered saline and immediately assessed by flow cytometry (Cytomics FC 500; Beckman Coulter, Hialeah, FL, USA). Data were analyzed using CXP Cytometer Software. Data were obtained from 10,000 events per sample. Numbers of Tregs were calculated based on the corresponding numbers of lymphocytes.

### 2.4. Statistical Analysis

The Mann–Whitney U test was used to assess differences in frequency of INS relapse, age, urinary protein, serum albumin level, and observation period. The χ^2^ test was used to assess differences in gender, daily intake at the time of INS onset, and use of immunosuppressive drugs. Median and interquartile ranges (IQRs) were calculated.

The gut microbiota of pediatric INS patients before and after probiotic treatment were compared with the gut microbiota of healthy children using the Kruskal–Wallis test. Changes in the Treg count were also analyzed by the Friedman’s test both in the probiotic-treated group and the non-probiotic-treated group. The Wilcoxon signed-rank test was used to compare percentages of butyrate-producing bacteria before and after probiotic treatment in children with INS.

Values of *p* < 0.05 were considered statistically significant.

## 3. Results

### 3.1. Characteristics of INS Patients

Table 1 shows the characteristics of the INS patients included in this study. Age, sex, observation period, urinary protein–creatinine ratio, and serum albumin levels at INS onset were not significantly different between the probiotic-treated and non-probiotic-treated groups. The frequency of consumption of dairy products such as cow’s milk, yogurt, and cheese (which may affect the gut microbiota [20]) was also investigated using a questionnaire given to guardians. There was no significant difference in consumption of dairy products between the two groups. In the probiotic-treated group, adherence to oral administration of CBM was also assessed via guardian surveys. Administration was confirmed for a median period of 25 months (range: 7 months to 46 months). The annual frequency of INS relapse was significantly lower in the probiotic-treated group (during probiotic treatment) than in the non-probiotic-treated group (within 2 years of the start of INS remission). In calculating the number of relapses per year, only relapses during the CBM interventional period were considered. The median number of relapses per year was 1.0 (IQR: 0–1.5 relapses) in the probiotic-treated group and 1.8 (IQR: 1.5–2.0 relapses) in the non-probiotic-treated group (*p* = 0.016). The number of patients treated with rituximab during the observation period was significantly lower in the probiotic-treated group (2 of 10 patients) than in the non-probiotic-treated group (7 of 10 patients) (*p* = 0.025). The median total dose of prednisolone in the probiotic-treated group (180.2 mg/kg) was less than that in the non-probiotic-treated group (270.9 mg/kg), although the difference was not statistically significant (*p* = 0.06).

### 3.2. Composition of the Gut Microbiota in Pediatric INS Patients before and after Probiotic Treatment

**Figure 1:**
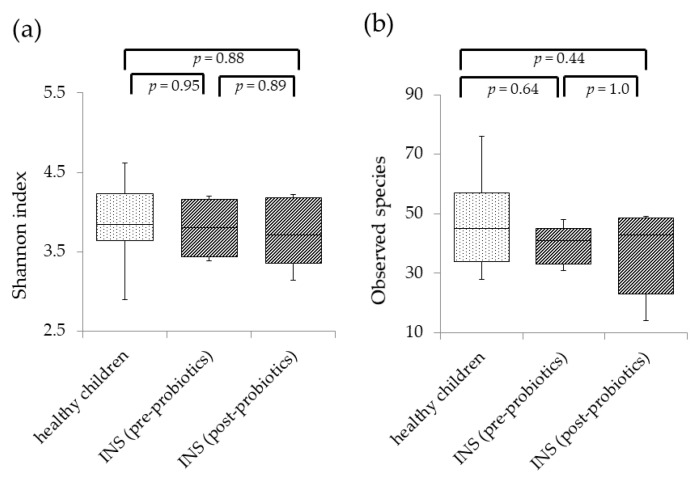
Analysis of the diversity of gut microbiota in healthy children and children with idiopathic nephrotic syndrome (INS) before and after *Clostridium butyricum* MIYAIRI administration. A whisker-plot diagram of Shannon index (**a**) and number of observed species (**b**) is shown for each group. Shannon index and the number of observed species were calculated in healthy children as well as children with INS before and after probiotic treatment. There were no significant differences among the three groups in either Shannon index or number of observed species. The central horizontal line in the box represents the median value, while the bottom and top edges of the box represent the first and third quartiles, respectively. Central vertical lines extend from the box to the 90th and 10th percentiles.

**Figure 2:**
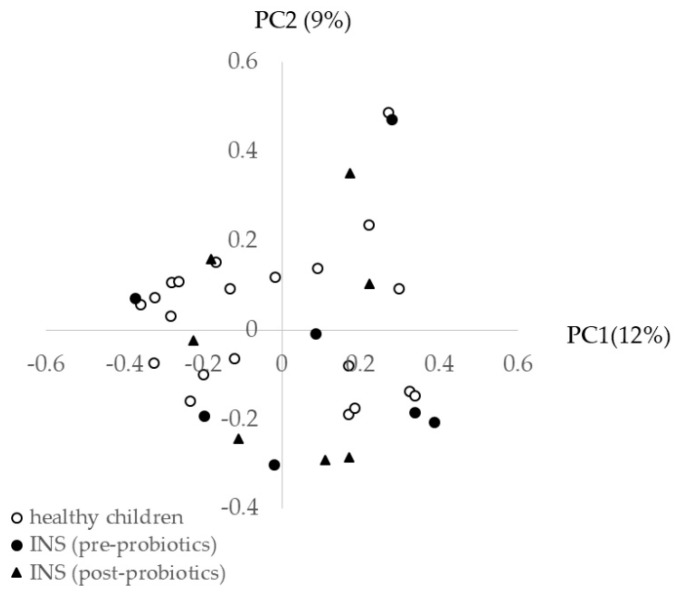
Principal coordinate (PC) analysis of the gut microbiota of healthy children and children with idiopathic nephrotic syndrome (INS) before and after probiotic treatment. There were no differences between the three groups. There was also no difference in clusters before and after probiotic treatment.

Figure 1 shows the diversity of the gut microbiota before and after probiotic treatment in INS patients compared with healthy children. In INS patients, there was no significant difference in the Shannon index (Figure 1a) or the number of observed species (Figure 1b) before and after probiotic treatment compared with healthy children. Principal coordinate analysis (PCoA) of healthy children before and after probiotic treatment was performed to characterize the samples on a two-dimensional surface. The PCoA plot showed that the three groups did not form independent clusters (Figure 2).

**Figure 3:**
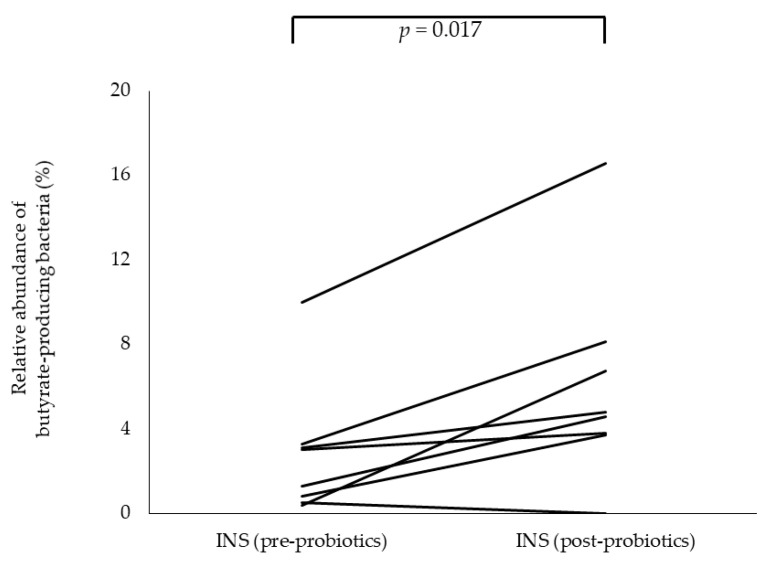
Changes in the composition of butyrate-producing bacteria in children with idiopathic nephrotic syndrome (INS) before and after *Clostridium butyricum* MIYAIRI administration. Analysis before and after probiotic treatment revealed a significant increase in the relative abundance of butyrate-producing bacteria at the species level.

Butyric acid produced by the gut microbiota is known to be essential for the differentiation and induction of Tregs. Therefore, we calculated the proportion of the following 19 known species of butyrate-producing bacteria among the 219 species detected by 16S rRNA gene sequencing: *Alistipes putredinis*, *Eubacterium ventriosum*, *Roseburia hominis*, *Anaerostipes caccae*, *Subdoligranulum variabile*, *Eubacterium rectale*, *Roseburia inulinivorans*, *Fusobacterium mortiferum*, *Fusobacterium nucleatum*, *Eubacterium hallii*, *Faecalibacterium prausnitzii*, *Clostridium symbiosum*, *Clostridium perfringens*, *C. butyricum*, *Megasphaera micronuciformis*, *Roseburia intestinalis*, *Odoribacter splanchnicus*, *Eubacterium limosum*, and *Anaerotruncus colihominis* [21]. The percentage of butyrate-producing bacteria was significantly lower before probiotic treatment (2.2% at INS onset) compared with that in healthy children (6.7%; *p* = 0.024). However, the percentage of butyrate-producing bacteria increased significantly after probiotic treatment (4.7%; *p* = 0.017) (Figure 3). *C. butyricum* (conspecific to the administered CBM) was detected in two of the eight patients.

### 3.3. Changes in Numbers of Tregs after Oral Probiotic Treatment in Children with INS

The number of Tregs at onset of INS was not different between the probiotic-treated group (median 57.0 cells/µL, IQR 45.5–66.7 cells/µL) and the non-probiotic-treated group (median 78.1 cells/µL, IQR 55.6–95.8 cells/µL) (*p* = 0.095). Oral CBM was started after INS remission in the probiotic-treated group and Tregs were measured at least twice before and after the start of CBM administration (*n* = 8). The median interval between blood sampling was 1.5 months (range: 1.2–2.6 months). The reason for the median 1.5-month period between initiation of CBM administration and stool/blood sample collection was that we aimed to collect samples prior to administration of immunosuppressive drugs, including prednisolone, before relapse.

**Figure 4:**
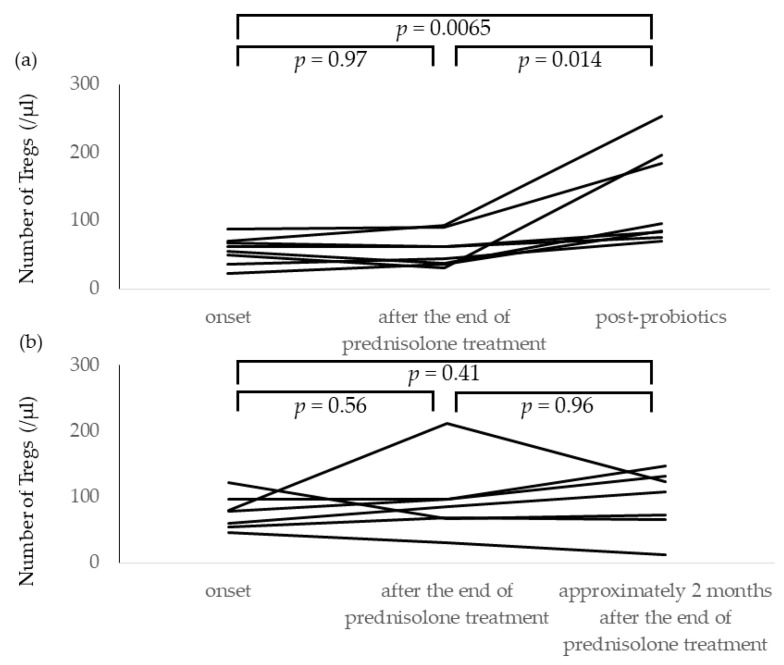
Changes in Treg frequencies after administration of *C. butyricum* MIYAIRI as a probiotic to patients with INS. Treg frequencies were measured at three time points: at the onset of INS, immediately after the end of prednisolone administration following induction of initial remission, and 1.5 to 2 months after starting probiotic administration following prednisolone cessation. (**a**) Probiotic-treated group: There was a significant increase in the number of Tregs after comparing with before probiotic treatment. (**b**) Non-probiotic treatment group: in the seven patients in the non-probiotic treatment group, there was no statistically significant increase in the number of Tregs at onset, immediately after the end of prednisolone treatment, and approximately 2 months after the end of prednisolone treatment.

Treg counts were significantly increased after CBM administration in the probiotic-treated group (Figure 4a). In contrast, as shown in Figure 4b, in the seven patients of the non-probiotic treatment group, there was no statistically significant increase in the number of Tregs at onset (median 78.1 cells/µL, IQR 55.6–95.8 cells/µL), immediately after the end of prednisolone treatment (median 85.2 cells/µL, IQR 67.5–96.1 cells/µL), and approximately 2 months (median 1.9 months, range 1.5–2.3 months) after the end of prednisolone treatment (median 108.4 cells/µL, IQR 69.3–127.4 cells/µL).

## 4. Discussion

Although the pathogenesis of pediatric INS is still unknown, we focused here on the potential immunological etiology of INS based on Shalhoub’s hypothesis proposed in 1974. This hypothesis stated that INS was a disorder of T-cell function resulting in increased plasma levels of lymphocyte-derived permeability factor [22], and was supported by the absence of immune complexes in the glomeruli, the rapid response to steroid therapy, the association of INS with Hodgkin’s disease, and the observation that measles infection often induced remission of INS. The abrupt development of massive proteinuria and hypoalbuminemia that characterize INS were thought to result from increased permeability of the glomerular capillary wall following T-cell activation triggered by stimuli such as viral infection or allergens. Recently, there have been reports of quantitative or qualitative reductions in Tregs in INS patients [23,24,25,26,27,28,29]. We also reported that the number of Tregs at the onset of INS was lower than in healthy children [13]. Although the reason for this decrease in Tregs at the onset of INS was unclear, we focused our attention on the gut microbiota of INS patients because of mounting evidence that butyric acid produced by some intestinal bacteria is essential for differentiation of Tregs in the intestinal tract [14]. We examined the gut microbiota at onset of INS and found that butyrate-producing bacteria were significantly reduced in frequency compared with healthy children [9]. Therefore, we hypothesized that administration of butyrate-producing CBM as a probiotic to INS patients with reduced numbers of butyrate-producing bacteria might inhibit subsequent INS relapse. We found that treatment with CBM after induction of INS remission prevented INS relapse and decreased the use of immunosuppressive drugs compared with non-probiotic-treated patients.

*C. butyricum* is a butyrate-producing, Gram-positive anaerobic bacterium known to inhabit the intestinal tract of healthy animals and humans [30]. The *C. butyricum* strain MIYAIRI 588 was isolated from the soil by Dr. Chikaji Miyairi in 1933. A CBM preparation available in Japan is used as an anti-flatulent and was reported to be effective as an antidiarrheal agent in children after antibiotic use [19]. Recently, CBM has been reported to have therapeutic effects on various diseases, including reduction of lipogenesis through the bacterial wall components and metabolites such as butyric acid [31], enhancement of the therapeutic effect of allergen-specific immunotherapy on allergic rhinitis [32], improvement of nonalcoholic steatohepatitis by increasing butyric acid in the intestinal tract [33], and amelioration of depression-like behavior in mice [34]. Kashiwagi et al. showed that prophylactic administration of CBM increased Treg frequency in mice. Subsequently, they reported that prophylactic administration of this drug prevented weight loss in mice even after induction of enteritis [35]. We showed in the present study that the percentage of butyrate-producing bacteria among intestinal bacteria increased after administration of CBM to INS patients, and that Treg counts increased as well. We speculate that the reason for the increase in Tregs was the effect of CBM administration on dysbiosis of the intestinal microbiota, which resulted in increases of butyrate-producing bacteria to levels like those of healthy children. It has also been reported that administration of *C. butyricum* increases butyrate-producing bacteria frequency among the gut microbiota [36].

This study had several limitations. First, organic acids in stool before and after CBM administration could not be measured because of low stored fecal volume. However, our previous studies have shown that patients with reduced butyrate-producing bacteria also have reduced butyric acid in their stools [9]. Therefore, we hypothesized that changes in butyrate-producing bacteria in the current study reflected the amount of fecal butyric acid in the intestinal tract. Second, *C. butyricum* conspecific to the administered CBM was detected in only two patients. A potential explanation of this finding is the low percentage of CBM in the gut microbiota. Third, there were limitations in the analytical capability of the sequencer for 16S rRNA sequencing. Fourth, as paired stool samples were collected at onset of INS and post-treatment with prednisolone in the probiotic-treated patients, they might reflect the effects of prednisolone on the intestinal microbiota. In this regard, to evaluate the effects of prednisolone on the intestinal microbiota, stool samples should have been collected at onset of INS and after the end of prednisolone administration from the non-probiotic treated group as well. However, because Treg measurements were performed immediately before and after probiotic administration, and were thus not affected by immunosuppressive drugs, we believe that at least changes in the numbers of Treg in the blood of probiotic-treated patients most likely resulted from the effects of CBM. Furthermore, the finding that there was no significant increase in the number of Tregs approximately 2 months after the end of prednisolone treatment compared with the number of Tregs immediately after the end of prednisolone treatment in the seven patients in the non-probiotic treatment group also lends support that the effects of prednisolone on the number of Tregs would be negligible.

In conclusion, administration of CBM as a probiotic to INS patients effectively reduces INS relapse and the frequency of immunosuppressive drug use. This probiotic can improve dysbiosis at the onset of INS and increase the frequencies of both intestinal butyr-ate-producing bacteria and Tregs.

## Figures and Tables

**Table 1:** Characteristics of idiopathic nephrotic syndrome (INS) patients.

	Probiotic Treatment Group	Non-Probiotic Treatment Group	*p*-Value
No. of patients	10	10	
Age, years	6.4 (3.7–10.6)	4.7 (3.5–7.8)	0.85
Sex (male/female)	9/1	6/4	0.30
Observation period (months) *	42.5 (36.8–50.5)	52.5 (37.3–57.3)	0.73
Urinary protein, g/g Cr	9.7 (5.8–11.6)	13.8 (8.8–27.2)	0.19
Serum albumin, g/dL	1.0 (0.7–1.5)	1.3 (1.0–1.85)	0.44
Consumption of dairy foods ^†^	6 (60%)	7 (70%)	0.64
Confirmed period of probiotic compliance (months)	25 (7–46)	Not applicable	
Total dose of prednisolone (mg/kg)	180.2 (83.8–230.5)	270.9 (194.2–294.2)	0.06
No. of INS relapses per year	1.0 (0–1.5)	1.8 (1.5–2.0)	0.016
Use of immunosuppressive drugs			
Mizoribine	7 (70%)	7 (70%)	1.0
Cyclosporin	6 (60%)	9 (90%)	0.12
Rituximab	2 (20%)	7 (70%)	0.025

* The observation period in this study was a combination of both the withdrawal period and the CBM interventional period. ^†^ Consumption of dairy foods such as cow’s milk, cheese, and yogurt at least once a day. CBM, *Clostridium butyricum* MIYAIRI; Cr, creatinine; INS, idiopathic nephrotic syndrome. Data represent counts (%) or medians (interquartile ranges).
